# Renal Transplantation from a Deceased Donor with Polycystic Kidney Disease

**DOI:** 10.1155/2021/6711155

**Published:** 2021-08-16

**Authors:** Marina M. Tabbara, Giselle Guerra, Gaetano Ciancio

**Affiliations:** ^1^Department of Surgery, University of Miami Miller School of Medicine, Miami, FL, USA; ^2^Miami Transplant Institute, University of Miami Miller School of Medicine, Jackson Memorial Hospital, Miami, FL, USA; ^3^Department of Medicine, Division of Nephrology, University of Miami Miller School of Medicine, Miami, FL, USA; ^4^Department of Urology, University of Miami Miller School of Medicine, Miami, FL, USA

## Abstract

Renal transplantation is the gold standard treatment for patients with end-stage renal disease (ESRD) as it demonstrates improved long-term survival compared to patients who remain on renal replacement therapy. The widening gap between the demand and supply of organs warrants the expansion of donor criteria for renal transplantation. Kidneys with multiple cysts are often rejected for transplantation. Here, we present our recent experience of a 72-year-old patient with ESRD due to a biopsy-proven diabetic nephropathy who received a deceased donor kidney with adult polycystic kidney disease (APKD). At 31-month posttransplant, he had a serum creatinine of 1.6 mg/dL. Deceased donors affected by APKD should be considered an acceptable option for successful renal transplantation in select recipients, as well as an alternative kidney source to increase the donor pool.

## 1. Introduction

Kidney transplantation is the treatment of choice for patients with end-stage renal disease (ESRD) as it has been shown to provide a better quality of life and superior long-term survival over those who receive other renal replacement therapy [1]. However, with a widening gap between demand and supply of organs, there have been growing interest and success in using expanded criteria donor (ECD) kidneys, whether it is donation after brain death (DBD) or donation after cardiac death (DCD) [2]. This includes the consideration of utilizing donor kidneys that are often rejected for transplantation, such as those with multiple cysts [3]. Polycystic kidney disease (PKD) is an inherited disease characterized by progressive cystic changes that leads to deterioration of renal function and often results in ESRD. It is commonly encountered in adults as an autosomal dominant disease, while the rare autosomal recessive form is more prevalent in the pediatric age group [4].

Several cases have been reported which describe kidneys from deceased donors affected by adult polycystic kidney disease (APKD) which were successfully transplanted, remaining fully functional for a long period of time [5–7]. Here, we report our experience of a patient with ESRD who received a deceased donor kidney with APKD.

## 2. Case Presentation

The recipient was a 72-year-old male with a BMI of 29.5 kg/m^2^ and a history of type 2 diabetes for over 10 years, hypertension, and chronic hepatitis C, who was diagnosed with a biopsy-proven diabetic nephropathy in 2012. He had been on peritoneal dialysis for approximately one year prior to transplantation from a deceased donor kidney with adult polycystic kidney disease (APKD) in 2019. The recipient had no preexisting human leukocyte antibodies at either class I or class II on repeated screening. The recipient and his sister, who is a physician, were counselled in detail about the donor's diagnosis of APKD and the potential risk of complications, including the growth of the kidney and graft failure, before giving informed consent prior to surgery.

**Figure 1:**
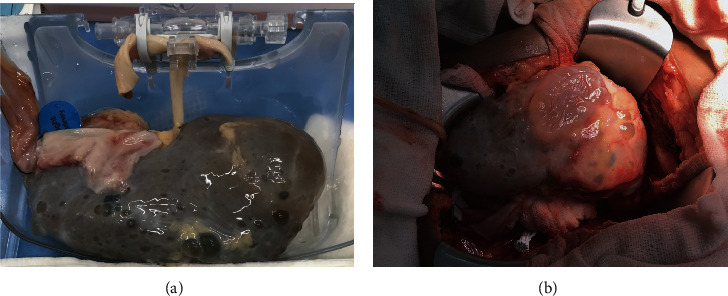
(a) Deceased donor polycystic right kidney prior to transplantation connected to the hypothermic machine perfusion; (b) deceased donor polycystic right kidney transplanted in the recipient's right iliac fossa.

The deceased brain death donor was a 40-year-old female with a BMI of 26.5 kg/m^2^ and a history of APKD who was admitted to the hospital following an intracranial hemorrhage secondary to left cerebral artery aneurysm rupture. The serum creatinine on admission was 0.76 mg/dL, and urine output was around 100 cc/hr. Predonation computed tomography scan showed numerous renal cysts bilaterally, with the largest measuring 5 cm on the right and 3.8 cm on the left. The right donor kidney was used for transplantation and measured 15 × 7 × 4 cm, showed multiple cysts, and had a single vein, artery, and ureter (Figures 1(a) and 1(b)). The donor's Kidney Donor Profile Index (KDPI) was 64%, which has been shown to have around a 75% graft survival rate at 5-year posttransplant [8]. The time zero biopsy (performed at the time of transplant) showed 4 glomeruli sclerosed out of 41 (9.7%), minimal chronic tubulointerstitial injury, minimal arteriolosclerosis, and mild acute tubular injury. The kidney was pumped on a hypothermic perfusion machine with a flow of 114 and resistance of 0.37 [9].

Our protocol induction therapy includes one dose of thymoglobulin 1 mg/kg, basiliximab 20 mg, and methylprednisolone 500 mg given intravenously at the time of implantation [10]. Our maintenance immunosuppression protocol includes a steroid-free regimen consisting of tacrolimus and mycophenolate mofetil starting on postoperative day 1. However, for this patient, it was changed to a maintenance immunosuppression consisting of mycophenolate mofetil, prednisone, and belatacept 5 mg/kg intravenous every month in efforts to protect the kidney from tacrolimus nephrotoxicity [11].

The recipient was discharged home on postoperative day 9 without surgical complications. Renal ultrasound at 4-month posttransplant reported the transplanted kidney that measured 15.5 cm in length with multiple cysts; the largest cyst measured 4.5 × 5.2 × 4.5 cm. At 31-month posttransplant, he had a serum creatinine of 1.6 mg/dL.

## 3. Discussion

Given the present shortage of donor kidneys, ECD kidney transplants have been accepted as lifesaving interventions for appropriate candidates. ECD kidneys from DBD and DCD have been instrumental in increasing the donor pool [12, 13]; however, donor kidneys that contain multiple cysts are still often rejected for transplantation [3].

According to published literature, polycystic donor kidneys are considered an acceptable option for transplantation, with some cases reporting normal renal function up to 15-year posttransplant [5, 6]. Graft survival of these kidneys has improved due to implementation of better immunosuppressive protocols that include the use of mTOR inhibitors and/or belatacept, which have been shown to decrease cyst enlargement and preserve kidney function [11, 14]. Kidneys from ECD donors are more susceptible to calcineurin inhibitor (CNI) nephrotoxicity [15]. A belatacept-based immunosuppression provides a beneficial effect in terms of renal function and long-term outcomes when compared to CNI-based regimens [11]. Improved graft survival is also based on the fact that there is sufficient renal reserve of the transplanted polycystic kidney that should allow it to function for an extended period, especially in older recipients [7, 16, 17]. Polycystic donor kidneys have provided comparable survival and quality of life benefit to the recipients as nonpolycystic donor kidneys; however, relatively short follow-up times and possible selection bias appear to keep these observations from being shown as statistically meaningful [7].

The natural history of progression to ESRD is variable in APKD, but it has been shown that 25% of all patients will develop renal failure by age 50 and 60% by age 70 [18]. It is important to note that there is approximately a 10-year lag period between the onset of symptoms and the progression to ESRD [16, 19]. This suggests that the age of deceased donors with APKD is crucial to prevent short-term failure and increased complications in the recipient [20].

When considering whether to transplant kidneys from expanded criteria donors, it is important that preimplantation biopsies are performed. Glomerulosclerosis of >20% is associated with poor graft outcome; however, this is only significant in specimens that included >25 glomeruli [21]. Additionally, the presence of vascular disease and arteriolar hyalinosis is a strong predictor of transplant failure [22].

Shamali et al. describe a comprehensive review of all published cases of polycystic donor kidneys between 1980 and 2018 and present 16 known cases. The median graft follow-up was 36 (range 6-180) months. The median serum creatinine level was 1.4  (range 0.84-4.99) mg/dL. They concluded that the use of APKD kidneys from younger deceased donors should be encouraged as an acceptable option to increase the donor pool for recipients that have been carefully selected by the transplant team [20].

Olsburgh et al. propose the following criteria in accepting donors with APKD: donors less than 50 years of age; kidney size less than 15 cm in length; normal creatinine level at the time of retrieval; a pretransplant renal biopsy that scores glomerulosclerosis, tubular atrophy, interstitial fibrosis, and vascular disease; a minimal cold ischemia preferably <12 hours but not >24 hours; and recipients with a life expectancy of 10 years or less [16]. In our case, the donor was a 40-year-old with an acceptable time-zero biopsy, except for a cold ischemic time of 35 hours due to travel time from St. Luke's University Hospital in Bethlehem, PA (Gift of Life Donor Program), to the Miami Transplant Institute.

In conclusion, APKD kidneys should not be automatically discarded but rather considered for transplantation in recipients who have a life expectancy of 10 years or less and if the donor is sufficiently young and has a smaller kidney size with preserved renal function and an acceptable time-zero biopsy.
